# Social Antecedents to the Development of Interoception: Attachment Related Processes Are Associated With Interoception

**DOI:** 10.3389/fpsyg.2019.00712

**Published:** 2019-04-24

**Authors:** Kristina Oldroyd, Monisha Pasupathi, Cecilia Wainryb

**Affiliations:** Social Development Laboratory, Department of Psychology, The University of Utah, Salt Lake City, UT, United States

**Keywords:** interoception, attachment, development, body awareness, interoceptive accuracy

## Abstract

Current empirical work suggests that early social experiences could have a substantial impact on the areas of the brain responsible for representation of the body. In this context, one aspect of functioning that may be particularly susceptible to social experiences is interoception. Interoceptive functioning has been linked to several areas of the brain which show protracted post-natal development, thus leaving a substantial window of opportunity for environmental input to impact the development of the interoceptive network. In this paper we report findings from two existing datasets showing significant relationships between attachment related processes and interoception. In the first study, looking at a sample of healthy young adults (*n* = 132, 66 males), we assessed self-reported interoceptive awareness as assessed with the Multidimensional Assessment of Interoceptive Awareness (Mehling et al., 2012) and attachment style as assessed with the Experiences in Close Relationships Scale-Short (Wei et al., 2007). We found relationships between aspects of interoception and attachment style such that avoidant individuals reported lower interoceptive functioning across several dimensions [*r*’s(130) = -0.20 to -0.26, *p*’s < 0.05]. More anxious individuals, on the other hand, reported heightened interoceptive across several dimensions [*r*’s(130) = 0.18 to 0.43, *p*’s < 0.05]. In the second study, we examined the congruence between a youth’s self-reported negative emotion and a measure of sympathetic nervous system arousal (SCL). The congruence score was positively associated with parental rejection of negative emotion. These results suggest that parenting style, as reported by the mother, are associated with a youth’s ability to coordinate their self-reported emotional and physiological responding across a series of independent assessments, *r*(108) = -0.24, *p* < 0.05. In other words, the more maternal reported parental rejection of youth negative emotions, the less congruent a youth’s self and physiological reports of distress.

## Introduction

### The Development of Interoceptive Functioning

Interoception refers to an individual’s ability to detect and track internal bodily cues (Garfinkel et al., 2015) and has been demonstrated to have important implications for psychological and physical health (Craig, 2004; Pollatos and Schandry, 2008; Paulus et al., 2009; Füstös et al., 2012; Herbert and Pollatos, 2014; Stern, 2014). While the literature is steadily revealing some of the biological underpinnings of interoception (Craig, 2004;Li et al., 2016), the social antecedents to interoception have been largely ignored. In this paper we propose that the development of interoception may be influenced by attachment related processes.

### A Brief Overview of Attachment Theory and the Importance of Attachment Related Processes

Bowlby (1982) posited that human infants develop attachment bonds with their caregivers. These bonds are characterized by specific patterns of cognition and behavior in children that influence a range of functioning from emotion regulation to how they experience their close relationships (Fraley and Roisman, 2018). When a child feels loved, secure and confident in their relationship with their caregiver, they will use the caregiver as a “secure base” from which to explore their environment (Ainsworth et al., 2015). These children can manage anxiety with some degree of trust and are able to use others to help resolve unpredictable, threatening, or novel life events (Bowlby, 1973; Bretherton, 1985; Ainsworth et al., 2015). An infant is most likely to develop a secure attachment when a parent consistently provides sensitive and attentive caregiving (Bowlby, 1973; Bretherton, 1985; Ainsworth et al., 2015).

When a parent avoids responding to the child’s immediate needs, makes them wait for relief and comfort, or responds frighteningly or inconsistently to their needs, children may develop an avoidant attachment (Ainsworth et al., 2015). Individuals with an avoidant attachment feel the need to be self-reliant, and emotionally strong, as others are perceived as only conditionally available (Hazan and Shaver, 1987). They tend to be rather isolated and place tremendous value on being independent. Avoidant individuals become enraged or highly anxious when forced to rely on others for help (Main et al., 1985; Dozier and Kobak, 1992).

An anxious attachment style often develops from the belief that the parent is available, but only conditionally, and that the parent is likely to withdraw that comfort and support if the child no longer meets certain standards – such as being well behaved or co-operative (Ainsworth et al., 2015). Individuals with an anxious attachment style tend to be overly clingy and become excessively upset when separated from their mothers or significant others (Hazan and Shaver, 1987).

While Bowlby was primarily focused on understanding the nature of infant-caregiver relationships, a plethora of research demonstrates that attachment characterizes human experience “from the cradle to the grave” (Bowlby, 1979, p. 129) (see Fraley and Roisman, 2018 for a review). From this perspective, attachment style affects more than just interpersonal functioning in infancy; it has enduring implications throughout the lifespan on emotion regulation, parenting practices, and health-related behaviors (Feeney and Collins, 2001; Waters and Waters, 2006; Raby et al., 2017). The present paper expands on these findings and introduces the idea that attachment related processes may have implications for the development of interoceptive functioning as well.

### Attachment Related Processes Influence a Person’s Physiological Response Patterns

While neuroanatomy provides the hardware necessary for interoception, the strength of the signal produced by the body is also important. In some individuals, there may be a stronger/weaker interoceptive signal available to detect. The strength of the interoceptive signal produced and the ease with which this signal is transmitted from sensory modalities to the interoceptive centers of the brain may depend upon HPA axis functioning. A large body of extant work links HPA axis functioning attachment related processes.

### Attachment and HPA Axis Functioning

Strong evidence exists that individual differences in attachment are characterized by differential HPA reactivity to stress (Allen and Miga, 2010; Diamond and Fagundes, 2010). In general, individuals with anxious and avoidant attachment styles exhibit dysregulated HPA axis activity in response to stress across the lifespan (Bush et al., 2011; Hill et al., 2011; Browne and Jenkins, 2012; Hackman et al., 2012; Lovallo, 2013; Palmer et al., 2013). Given that stress and interoceptive functioning utilize the same anatomical pathways to facilitate communication between the brain and the body, attachment related processes that affect the stress response system (the descending brain-body connection) could also affect the interoceptive system (the ascending brain-body connection) (Seth, 2013). We believe that dysregulation of the HPA axis could affect interoception in two ways: by affecting the strength of the interoceptive signal and/or by affecting the processing of the interoceptive signal.

One example of an interoceptive signal that changes with HPA axis functioning is the stroke volume of the heart, defined as the amount of blood pumped by the heart in one contraction (Schächinger et al., 2001). The activation of the HPA axis results in the release of several hormones, including epinephrine. Epinephrine causes increased contractibility of the heart muscle, increased heart rate and increased depolarization of the heart, all of which lead to an increase of stroke volume (SV). Increased stroke volume has been empirically associated with increased interoceptive accuracy (Schandry et al., 1993) such that the more blood the heart pumps per beat, the better people are at estimating the number of times their heart beats during a timed trial. Thus, increased SV is thought to function as a “stronger” interoceptive signal. A chronically increased sympathetic outflow has been suggested to be one variable contributing to the establishment of high interoceptive accuracy (Paulus and Stein, 2010).

In addition to changing the strength of the signal, increased HPA activation may affect interoception by changing how the interoceptive signal is processed. Cortisol, the final product of HPA axis activation, has been shown to modulate interoceptive signal processing such that the brain becomes increasingly attuned to interoceptive signals in its presence (Rief et al., 1998). For example, a dose of 4 mg of intravenously administered cortisol has been demonstrated to increase performance on tests of Interoceptive Accuracy (IAcc) (Schulz et al., 2013). These data suggest that cortisol may lower the threshold for interoceptive signal processing within the brain (Schulz et al., 2013). This finding is supported by fMRI data indicating that the parts of the brain responsible for the attentional processing of interoceptive signals (e.g., the ACC and OFC) show greater activation in the presence of cortisol (Cameron, 2002; Critchley et al., 2004; Pollatos et al., 2007). Thus, when the HPA axis is activated it may alter how the brain deals with incoming bodily cues (Craig, 2002; Schulz and Vögele, 2015). If a person experiences chronic dysregulation of the HPA axis, this may permanently induce altered perception of bodily cues (Schulz and Vögele, 2015).

### The Interoceptive Network of the Brain Is Affected by Attachment Related Processes

The basic architecture of the brain is constructed through an ongoing process that begins before birth and continues into adulthood (e.g., Goddings and Giedd, 2014). Attachment related processes have been demonstrated to affect the quality of that architecture (Cozolino, 2014). From this perspective, normal brain development, including development of the interoceptive network, may be dependent upon a satisfactory attachment (Emde, 1988; Schore, 2000).

The interoceptive network is composed of three major brain regions: the anterior insula cortex (AIC), the anterior cingulate cortex (ACC) and the orbitofrontal cortex (OFC) (Craig, 2002, 2009; Kurth et al., 2010; Uddin, 2015). Each of these regions may be influenced by attachment related processes in ways that could be important for the development of interoception. For example, the AIC is the interoceptive center of our brain (Craig, 2004). Attachment related processes have been found to be correlated with insular anatomy such that children who are classified as having an anxious or an avoidant attachment style demonstrate markedly lower insular volume and smaller surface area than control groups (Kühn and Gallinat, 2013; Sheffield et al., 2013; Lim et al., 2014). Additionally, attachment related processes have been shown to be related to electrical activation in the insula such that people with an avoidant attachment style showed decreased insular activation in response to stimuli than do securely attached individuals (DeWall et al., 2011).

### The Development of a Bodily Self and Interoception

Lastly, we believe that attachment related processes may influence the development of interoception by influencing the development of the bodily self (Gallagher, 2000; Tsakiris, 2017). Because young infants have limited resources, they cannot use action to collect evidence about the causes of their own interoceptive experiences. Instead, infants rely on caregivers’ reactions to their behaviors to inform conceptualizations of interoceptive states. For example, when a young infant becomes fussy they may not understand the source of their own discomfort. It is only when a caregiver provides the child with a nipple and the act of eating begins to alleviate the infant’s discomfort, does the infant start to learn about the feeling of hunger. Insensitive caregiving, characterized by slow or intermittent responsiveness to the infant’s needs and rejection of infant distress may impair the child’s ability to form accurate representations of bodily sensations.

### Summary

To summarize, thus far we have outlined the idea that links may exist between attachment related processes and the development of interoception. Attachment may influence the development of interoception by modifying functioning of the HPA axis, by affecting the growth of neural architecture, and by influencing development of the bodily self. In Study 1 we examine whether attachment style is correlated with an individual’s interoceptive functioning in a sample of young adults. In Study 2 we consider how parenting style may be related to a youth’s coordination of physiological and self-reported aspects of emotional distress.

## Study 1: Introduction

Attachment related processes may lay the foundation for the development of interoception. Just as different attachment styles are associated with distinct behavioral responses to interpersonal cues, that attachments styles will also be associated with distinct behavioral responses to bodily cues. Thus, people with an anxious attachment who exaggerate the seriousness of relationship threats, over-emphasize their sense of helplessness and vulnerability in relation to their partners (Mikulincer and Shaver, 2012), and overly attend to internal indicators of emotional distress (Cassidy and Kobak, 1988) may respond similarly to bodily cues. This would include paying hypervigilant attention to the bodily sensations. By contrast, those high in attachment avoidance, who tend to minimize experiences of negative affect and to direct attention away from threat cues in interpersonal situations (see Diamond and Fagundes, 2010 for a review), may divert attention from bodily cues, suppress emotion-related action tendencies, or inhibit and mask bodily cues.

Traditional methods of assessing interoception focus solely on a person’s ability to accurately detect bodily cues such as the heartbeat (e.g., Whitehead et al., 1977; Schandry, 1981). However, we believe the concept of interoception to be a more nuanced concept than the simple ability to track heartbeats. The Multidimensional Assessment of Interoceptive Awareness Scale (MAIA) was developed in order to allow researchers to assess a person’s attitudes and beliefs about their bodily cues and to parse beneficial from maladaptive functions of interoception (Mehling et al., 2012). The MAIA consists of eight subscales: not-worrying, emotional awareness, attention regulation, trusting, body listening, noticing, not-distracting, and self-regulation. Preliminary research suggests that each of these subscales are associated with distinct neural patterns of activation in the interoceptive network (Stern et al., 2017). These patterns of neural activation may underlie discriminate behavioral responses to bodily cues: hyperarousal for individuals with an anxious attachment and hypoarousal for individuals with an avoidant attachment. In study 1 we examine correlations between self-reported attachment style and self-reported interoceptive functioning. We predict that self-reported attachment style will be associated with the following patterns of scores:

H1a: Avoidant individuals will manifest lower scores of the subscales of noticing, not distracting, not worrying, emotional awareness, body-listening and trusting.
H1b: Avoidant individuals will score higher on the attention regulation scale.
H1c: Anxious individuals will have higher scores on noticing, emotional awareness, body listening.
H1d: Anxious individuals will show lower scores on the not-distracting, not-worrying, self-regulation and attention regulation subscales.

## Study 1 Methods

### Participants

This study made use of existing data from previously conducted research (Oldroyd et al., unpublished) designed to examine the link between embodied narration and caloric consumption. Participants were 135 students (68 male) drawn from the participant pool at a large Rocky Mountain university. The average age was 23.5 years (*SD* = 5.9). The majority of participants identified as white (77%) with others identifying as Asian (17%), Pacific Islander (0.05%), Black (0.02%) and Latino (0.02%). 5% of participants chose to not report.

### Procedure

Upon arriving at the lab, participants provided written informed consent. Next, participants played a video game on an iPad, wrote narratives about their experience playing the video game, and then completed a 20-min questionnaire session during which time they had *ad libitum* access to snack foods. The order of the questionnaires was randomized, with the exception of the demographics questionnaire and a questionnaire that asked participants about the snack foods that they had been offered. These were presented last, so as to not influence participants’ eating behaviors. Upon completion of the questionnaires, participants were debriefed and dismissed.

### Measures

Twelve questionnaires were administered: Five of these questionnaires were theoretically important to the questions being asked in the original study examining the effect of embodied narration on caloric consumption (Oldroyd et al., unpublished). They were the MAIA, ECR-S, NASA-TLX, Emotional Responding, and Barrett Impulsivity Scale. Two of these questionnaires were of theoretical importance to the questions presented in this paper and will be discussed in detail below. They are the Multidimensional Assessment of Interoceptive Awareness (Mehling et al., 2012) and the Experiences in Close Relationships—Short (Wei et al., 2007). The other questionnaires were time fillers that were given in order to extend the period of time that our participants spent in the lab and had snack foods available to them. All of the questionnaires listed were scored and an ANOVA run on each one to make sure that scores did not differ by assigned narrative condition. Results for the two key questionnaires are as follows: MAIA: *F*(3,129) = 11.96, *p* = 0.41 and ECRS: *F*(3,131) = 42.20, *p* = 0.94.

#### Measures Used in This Study

##### Multidimensional assessment of interoceptive awareness (MAIA)

The MAIA is a multifaceted body awareness questionnaire that is designed to measure interoceptive awareness. The MAIA is composed of 32 items on a 6-points Likert scale, with ordinal responses coded from 0 (“never”) to 5 (“always”). This multidimensional instrument results in eight subscales: (1) Noticing, the awareness of one’s body sensations (4 items, Cronbach’s α = 0.64); (2) Not-distracting, the tendency not to ignore or distract oneself from sensations of pain or discomfort (3 items, Cronbach’s α = 0.64); (3) Not-worrying, the tendency not to experience emotional distress or worry with sensations of pain or discomfort (3 items, Cronbach’s α = 0.69); (4) Attention regulation, the ability to sustain and control attention to body sensation (7 items, Cronbach’s α = 0.73); (5) Emotional awareness, the awareness of the connection between body sensations and emotional states (5 items, Cronbach’s α = 0.73); (6) Self-regulation, the ability to regulate psychological distress by attention to body sensations (4 items, Cronbach’s α = 0.72); (7) Body listening, the tendency to actively listen to the body for insight (3 items, Cronbach’s α = 0.65); and (8) Trusting: the experience of one’s body as safe and trustworthy (3 items, Cronbach’s α = 0.74). The score for each scale is calculated by averaging the scores of its individual items, and thus can vary in the 1–5 range.

##### Experiences in close relationships-short (ECR-S)

Attachment anxiety and avoidance were assessed using the 12-item short version of the Experience in Close Relationships (ECR-S; Wei et al., 2007). The ECR-S has two 6-item subscales evaluating people’s attachment anxiety (e.g., “I worry that romantic partners won’t care about me as much as I care about them”) and avoidance (e.g., “I try to avoid getting too close to my partner”). Each of the 12 items was scored on a 7-point scale ranging from 1 (*disagree strongly*) to 7 (*agree strongly*). Low scores on both anxiety and avoidance represent attachment *security*. Cronbach’s α = 0.72 for the Anxiety Scale and Cronbach’s α = 0.74 for the Avoidance Scale.

#### Measures Collected but Not Used in This Study

The following questionnaires were administered during the course of the original study. They were not of theoretical interest to either the original study or to his one. They were instead used as time fillers to extend the amount of time that participants spent in the lab and with the proffered snack foods. These questionnaires are: Berkeley Expressivity Questionnaire (Gross, 2013), Basic Needs Scale (Johnston and Finney, 2010), Flourishing Scale, (Diener et al., 2010), Ryff Scale of Well Being (Ryff, 1989), Big Five Personality John and Srivastava, 1999, demographic questionnaire, and snack questionnaire.

## Study 1 Results

Correlations between the subscales of the MAIA are reported in Table 1. Descriptive statistics of the subscales of the MAIA are reported in Table 2. Tests of the a priori hypotheses were conducted using Bonferroni adjusted alpha levels. Correlations between the attachment style and self reported interoception indicated that individuals that score high in attachment anxiety also tend to score high on the noticing scales, *r*(133) = 0.18, *p* < 0.05, and on the emotional awareness scale, *r*(133) = 0.18, *p* < 0.05. See Table 3. Individuals who score higher in attachment anxiety also manifest a negative correlation with the ‘not-worrying’ scale, *r*(133) = -0.43, *p* < 0.001, indicating that the more anxious a person’s attachment style, the more they notice and worry about their bodily cues.

**Table 1:** Pearson correlation matrix among subscales of the MAIA.

		Not	Not	Attention	Emotional	Self	Body
	Noticing	distracting	worrying	regulation	awareness	regulation	listening	Trust
Noticing	1	0.09	-0.01	0.45**	0.52**	0.39**	0.45**	0.31**
Not distracting	0.09	1	-0.01	-0.04	0.01	-0.03	0.01	-0.05
Not worrying	-0.01	-0.01	1	0.32*	-0.02	0.31*	0.13	0.36**
Attention regulation	0.45**	-0.04	0.32**	1	0.31**	0.57**	0.50**	0.58**
Emotional awareness	0.52**	0.01	-0.02	0.31**	1	0.32**	0.48**	0.32**
Self regulation	0.39**	-0.03	0.31**	0.57**	0.32**	1	0.36**	0.46**
Body listening	0.45**	0.01	0.13	0.50**	0.48**	0.36**	1	0.45**
Trust	0.31**	-0.05	0.36**	0.58**	0.32**	0.46**	0.45**	1


*^∗^p < 0.05, ^∗∗^p < 0.01*.

**Table 2:** Descriptive statistics for the MAIA and ECRS (*N* = 135).

	Minimum	Maximum	Mean	*SD*
Noticing	1	5	3.74	0.63
Not distracting	1	3	1.73	0.45
Not worrying	1	5	2.24	0.76
Attention regulation	1	5	3.39	0.56
Emotional awareness	2	5	4.00	0.56
Self regulation	2	5	3.66	0.70
Body listening	1	5	3.32	0.73
Trust	1	3	1.93	0.34
MAIA total score	20	38	29.35	3.46
ECRS anxious	8	39	23.07	6.43
ECRS avoidant	6	37	19.80	6.43


**Table 3:** Pearson correlation matrix among subscales of MAIA and attachment style.

	Noticing	Not distracting	Not worrying	Attention regulation	Emotional awareness	Self regulation	Body listening	Trust
ECRS Anxious	0.18*	-0.05	-0.43**	-0.05	0.18*	-0.12	0.05	-0.09
ECRS Avoidant	-0.14	-0.10	-0.03	-0.20*	-0.04	-0.13	-0.12	-0.26**


*^∗^p < 0.05, ^∗∗^p < 0.01*.

**FIGURE 1:**
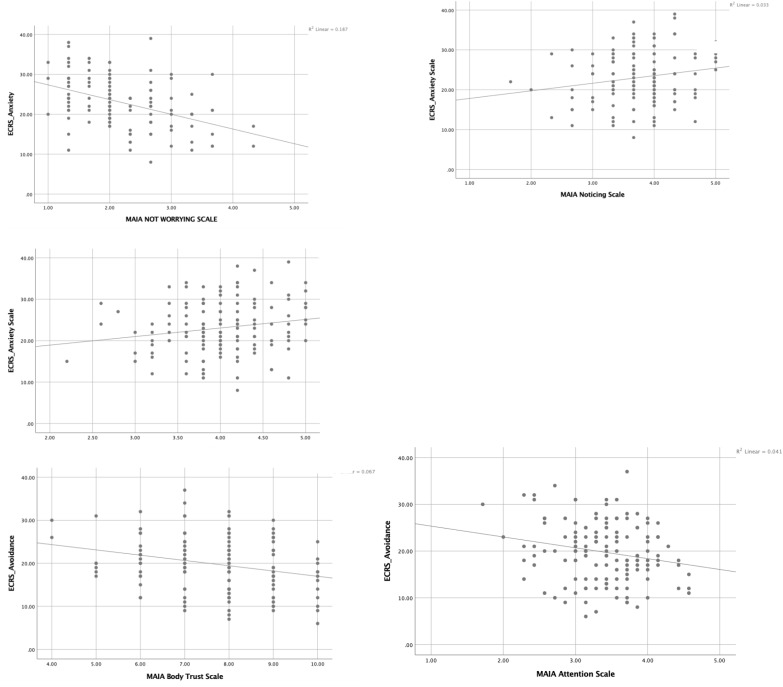
Scatterplots of significant correlations from Study 1.

Individuals who scored high in attachment avoidance scored lower on the scale of attention, *r*(133), -0.20, *p* < 0.05 and trust, *r*(133) = -0.26, *p* < 0.001. This means that the more avoidant a person’s attachment style, the less attention they paid to their bodily cues and the less they tended to trust those cues. Scatterplots of the data are presented in Figure 1.

## Study 1 Discussion

These findings offer support for the idea that attachment related processes and interoceptive functioning are correlated and suggest that people’s responsivity to bodily cues may mirror their responsivity to interpersonal cues. For example, individuals with an anxious attachment style who often demonstrate a hyper reactivity to social/relationship stimuli, are vigilant in detecting potential interpersonal threats, persistently signal their distress, and seek excessive reassurance/support in social situations (Noriuchi et al., 2008; Vrticka and Vuilleumier, 2012), may repeat this pattern of behavior with regards to bodily cues. This would explain the positive correlation between an anxious attachment and the noticing subscale. For these individuals, hypervigilance may manifest as excessively attentive monitoring of bodily sensations for threat. Attentional vigilance for bodily symptoms results in a greater chance of detecting potential sources of threat, exacerbating pain, deterioration in physical health, and social isolation (Salkovskis and Kobori, 2015). These individuals may misinterpret normal body symptoms as an indicator of a serious or threatening health problems. This would account for the negative correlation between anxious attachment and the ‘not-worrying’ subscale.

Avoidant individuals have often been described in the psychophysiology literature as manifesting a disconnect between their bodily cues and their physiological responses (e.g., Diamond et al., 2006). For example, the person with an avoidant attachment disorder may present as if they are very calm while in a distressing situation, when in fact psychophysiological measures show an elevated heart rate and cortisol levels (Spangler and Grossmann, 1993; Diamond and Fagundes, 2010). Over time, avoidant individuals learn to suppress their behavioral responses and to overregulate their affect, resulting in the appearance that they are unaffected by stressful situations. Given this, the negative correlation between body attention and avoidant attachment style makes sense. In the face of potentially negative bodily cues, avoidant individuals may minimize, dismiss and suppress them (as they do with problematic social cues) (Fraley and Shaver, 1997; Allen et al., 1998). This is in line with the notion that avoidant individuals are ‘preemptive’ in the avoidance of stress such that they disengage their attention from potentially distressing experiences before negative affect has been encoded and experienced (Fraley et al., 2000).

In addition to displaying decreased negative affect, avoidant individuals also demonstrate lower levels of trust: trust in close, personal relationships (Mikulincer, 2004) and trust in themselves (Cassidy, 2001). The negative correlation between body trusting and avoidant attachment follows this pattern. Thus, a person who has not developed trust in a loving caretaker and learned to trust their own decisions about who is safe and who is not, may not expect their body to give them reliable and important signals that warrant their attention.

Although these data provide support for our proposition regarding the relation between attachment related processes and interoception, Study 1 was limited in that both variables of interest (e.g., attachment and interoception) were obtained via self-report questionnaires in a collegiate sample. A second set of available data in our laboratory allowed us to examine the extent to which individuals were coherent across physiological and psychological responding – a different take on interoception – and their mothers’ general responses to their emotional distress.

## Introduction to Study 2

Autonomic nervous system (ANS) activity reflects an awareness of and a responsiveness to the environment and supports behavioral and emotional regulation (Beauchaine, 2001; Porges, 2007). One parameter of ANS reactivity, skin conductance level (SCL), is considered to be an objective marker of the sympathetic branch of the ANS (Beauchaine, 2001). Thus, greater SCL responding is indicative of greater emotional arousal. Recent work suggests that positive correlations between subjective and objective reports of arousal are associated with greater activity in the interoceptive network of the brain such that, the more congruence between a person’s self-reported emotional arousal and SCL activity, the better his interoception functioning may be (Kleckner et al., 2017). In Study 2 we examine the congruence between youth’s self-reported emotional arousal and SCL arousal and consider how attachment related processes may be related to a child’s congruence scores.

Caregiving is an example of an attachment related process, and empirical work shows that the attachment and caregiving systems are often activated simultaneously (Doinita and Maria, 2015). Thus, the type of care that a child receives can be predictive of the type of attachment that they will form (George and Solomon, 1999). Caregiving that is responsive and accepting is positively associated with a secure attachment style and has also been indicated as a precursor to the development of a core bodily self (Fotopoulou and Tsakiris, 2017). Thus, an infant that is “affectively attuned” with their caregiver (Stern, 1985) may develop stronger interoceptive abilities. From this perspective, the development of interoception is a generative model wherein a caregiver’s actions combine with an infant’s perceptions of bodily cues and the origins of core subjective feelings such as hunger and satiation, cold and warmth are social not biological (Fotopoulou and Tsakiris, 2017).

One hallmark of sensitive parenting is the parental acceptance of negative emotion. By contrast, insensitive parenting can include the rejection of negative emotion. Parental rejection of negative emotion occurs when caregivers reject, ignore, or fail to respond to a child’s signs of distress.

In Study 2 we examine the congruence between youth’s self-reports of negative emotional responding to an angry memory and physiological reports of negative emotional arousal as a function of mother’s caregiving style. According to attachment theory, children with mothers high in acceptance will feel more comfortable acknowledging their own distress and thus have greater congruence between their subjective reports and physiological manifestation of arousal. In contrast, admitting distress may be difficult for children with an avoidant attachment, who often adopt a deactivating, minimizing strategy toward negative cues. Given that dismissing children may have an interest in downplaying their distress, we would expect that they would report less emotional responding than their physiological responding would suggest. This would result in lower congruence scores. Thus, we hypothesized that youth with a mother who is more accepting of negative emotions will manifest a greater congruence score between self-reported distress and physiological measures of distress. Further, we present the idea that parental rejection of children’s negative emotions may also affect children’s development of interoception.

H2a: Mothers’ scores on a measure of parental rejection of negative emotion will be related to lower congruence scores between 0 and -1 in their children.
H2b: Mothers’ scores on a measure of parental acceptance of negative emotion will be related to higher congruence scores between 0 and 1 in their children.

## Study 2 Methods

### Participants

Participants in this study included 108 youth and their mothers. Youth (53 male) were drawn from a community of a medium-sized Rocky Mountain city. Participants ranged in age from 8 to 17 years old, evenly distributed across the age continuum. The majority identified as white (88%) with other participants identifying as Asian (1.5%), Pacific Islander (2.2%), Black (3.6%), Native American (2.9%), and Latino (5.8%). Mothers (*N* = 108) ages ranged from 27 to 61 years (*M* = 42.54, *SD* = 6.36). This was an educated sample with all of our mothers reporting that they had graduated high school, 27% completed some college, 45% having a college degree, and 18% having a post-graduate degree. The majority of participants identified as white (91%) with others identifying as Asian (1.5%), Pacific Islander (1.5%), Black (1.5%), Native American (2.9%), and Latino (4.4%).

Participants in this study were a subset of participants used in a larger NIH funded study on narration and emotion regulation (Wainryb et al., 2018). Participants were included in this study if they had been randomly assigned during the first study to the narrate condition. Limiting our study to those in the narrate condition standardized the experimental protocol, allowing us to examine the relevant questions for this study without the confounding variable of experimental condition. The original paper did not report on physiological data or on mothers’ questionnaire data.

### Procedure

Youths and their mothers arrived together and following assent/consent procedures wherein both participants completed written, informed consent, they were separated for the remainder of the study. Youth were taken into a private room and were hooked up to physiological recording equipment. Once the equipment was in place youth completed several baseline tasks including a 3-min vanilla baseline, an easy task designed to relax and orient the participant to the lab environment while giving the equipment time to calibrate, a 2-min talking baseline which allowed us to get baseline physiological readings while the participant was conversing with a research assistant, and a 4-min paced respiration task designed to obtain baseline respiratory sinus arrhythmia (RSA) (Diamond and Otter-Henderson, 2007). Next, youth were asked to, “Think of time when someone said or did something and you ended up feeling really angry at that person.” Once a memory had been retrieved, youth were told to spend 3-min thinking about that memory (exposure). After a 1 min rest period, participants narrated their story to a trained research assistant (regulate). Following another 1 min rest period, participants were asked to think again about the nominated memory (re-exposure). Following each task, participants filled out a questionnaire to assess self-reported emotional responding. After re-exposure, the physiological equipment was removed. Participants completed a manipulation check, provided a title for their angry memory, and reported how long ago the memory occurred. Participants were then compensated for their time and excused. For full details on the experimental protocol see the original publication (Wainryb et al., 2018).

Following assent and consent procedures, mothers were placed in a room by themselves and asked to complete a computerized survey consisting of 11 questionnaires. Of interest to this study was the Emotion Related Parenting Questionnaire-Short (Gottman and DeClaire, 1997), which is described in detail below. Other questionnaires administered but not part of this study are listed in the Measures section below.

### Measures

#### Youth

##### Self-reported emotional responding when recalling and narrating about past events

Following each of the five tasks in the study (Vanilla baseline, talking baseline, exposure, regulation, re-exposure) participants reported the extent to which they felt angry, scared, ashamed, sad and guilty on a scale of 1 (not at all) to 7 (extremely). In recent physiological research, the vanilla baseline technique has replaced the resting baseline period with a simple, minimally demanding task to maintain consistent alertness and baseline stability (Jennings et al., 1992). Scores for all emotions were summed and divided by 5 to compute a “Negative Emotional Responding” variable for each task. Scores were standardized within person by finding the standard deviation of all five scores and then dividing the mean differences by standard deviation.

##### Skin conductance level (SCL)

Skin conductance level (SCL) was measured and analyzed using Biopac MP 150 system. Skin conductance was recorded continuously throughout each session, with task on and offsets also recorded. Average SCL was computed as an index of sympathetic nervous system arousal for each task. Scores were standardized within person by finding the standard deviation of all five scores and then dividing the mean differences by the standard deviation.

##### Self-report and physiological congruence score

For the youth participants, the primary measure of interest for this study was a congruence score between self-reported emotional responding and physiological measures of emotional responding. This congruence score was derived by computing the correlation between two measures: subjective emotional responding across the two baseline, exposure, regulation, and re-exposure epochs of the experiment and the physiological reports of sympathetic nervous system arousal during the same time periods. We correlated youth’s self-reported negative emotional responding with the task-average SCL reading across the five tasks: vanilla baseline, talking baseline, exposure, regulation, and re-exposure. Higher correlations are indicative of more congruence between a youth’s self-report and physiological responding. Thus, for a participant with a positive correspondence score, when their self-reported negative emotional responding increased, so did SCL. For a participant with a negative correspondence score, when SCL increased, self-reported emotional arousal decreased. Correlations in this sample ranged from *r* = -0.84 indicating that when participants’ SCL increased, self-reported emotional responding decreased, to *r* = 1.0 indicating that when SCL increased, self-reported emotional responding also increased.

#### Mothers

Mothers completed the *Emotion Related Parenting Scale*, *Short* (ERPS-S; Gottman and DeClaire, 1997), a 20-item questionnaire that results in four scale scores, each representing a different parenting style as described in meta-emotion theory (Gottman and DeClaire, 1997). Responses for each scale are summed and divided by the total number of items for that subscale. Each scale is comprised of three items. The resulting four scales are labeled (1) emotion coaching scale (Cronbach’s alpha = 0.73), (2) feelings-of-uncertainty/ineffectiveness scale (Cronbach’s alpha = 0.73), (3) parental rejection of negative emotion (Cronbach’s alpha = 0.64), and (4) parental acceptance of negative emotion scale (Cronbach’s alpha = 0.74). Measures obtained but not used in this study include: *Children’s Reports of Parental Behavior Inventory* (CRPBI-30; Schuldermann and Schuldermann, 1970), The Berkeley Expressivity Questionnaire (Gross and John, 1997), The 10-item Emotion Regulation Questionnaire (Gullone and Taffe, 2012), BRIEF, Strategies of Anger Regulation in Adolescents (SAR-C) (von Salisch and Vogelgesang, 2005), and about themselves: Strategies of Anger Reduction (SAR-A) (von Salisch and Vogelgesang, 2005), SARI (Sadness and Anger Rumination Index, Peled, 2006), Buss Perry aggression questionnaire (Buss and Perry, 1992), Big Five Inventory (Goldberg D. T., 1993; Goldberg L. R., 1993); Test of Self-Conscious Affect, TOSCA (Tangney, 1991), Experiences in Close Relationships, ECR-S (Wei et al., 2007).

## Study 2 Results

A multiple regression was run wherein mother’s scores on the parental acceptance of negative emotion and the parental rejection of negative emotion subscales of the *Emotion Related Parenting Scale*, *Short* (ERPS-S; Gottman and DeClaire, 1997) were entered as a predictor of a youth’s congruence score. Youth’s age and gender were also entered in the model. Correlations are reported in Table 4. *R*^2^ for the overall model was 8.4% with an adjusted *R*^2^ of 4.7%. The model was statistically significant, *F*(1,107) = 2.92, *p* = 0.05. Results indicate that a mother’s score on the subscale titled, ‘Rejection of Negative Emotion’ could significantly predict a youth’s congruence score, such that the more a mother endorses items indicating that she rejects her youth’s negative emotions, the less her youth’s self-reported emotional responding scores are congruent with their physiological measures of responding. The regression equation was: Congruence score = 0.33^∗^(-0.03) (Mother’s score on parental rejection of emotion scale). Regression coefficients and standard errors are reported in Table 5.

**Table 4:** Pearson correlation matrix among parenting styles and congruence scores.

	Congruence score	Emotion coaching	Rejection of negative emotion	Acceptance of negative emotion	Uncertain and ineffective parenting	Child age	Gender
Congruence	1	-0.01	-0.26**	0.16*	-0.04	0.17	-0.08
Emotion coaching	-0.01	1	-0.15	0.18*	-0.31**	-0.06	-0.12
Rejection of negative emotion	-0.26**	-0.15	1	-0.35**	0.53**	-0.16	0.18*
Acceptance of negative emotion	0.16*	0.18*	-0.35**	1	-0.16*	-0.05	0.08
Uncertain and ineffective parenting	-0.04	-0.30**	0.53**	-0.16*	1	-0.03	0.09
Child age	0.17	-0.06	-0.16*	-0.05	-0.03	1	-0.10
Gender	-0.08	-0.22*	0.19*	0.08	0.09	-0.10	1


*^∗^p < 0.05, ^∗∗^p < 0.01*.

**Table 5:** Summary of multiple regression analyses for variables predicting child’s congruence score (*N* = 108).

Variable	*B*	*SE B*	*β*
Parental rejection of negative emotion	-0.03	0.01	-0.21*
Parental acceptance of negative emotion	0.01	0.01	0.05
Age	0.02	0.01	0.13
Gender	-0.03	0.09	-0.04
*R*^2^	0.05		
*F*	2.92^∗^		


*^∗^p < 0.05*.

## Study 2 Discussion

Study 2 investigated whether attachment related processes, operationalized as parental acceptance or rejection of negative emotion, could predict congruence between youth’s objective and subjective measures of emotional responding. This congruence score operates as a measure –albeit imperfect – of interoceptive functioning (see Kleckner et al., 2017). We found that rejection of negative emotion decreased congruence between a youth’s objective and subjective measures of emotional responding.

The results of Study 2 support our hypotheses by demonstrating that the higher a mother’s score on the Rejection of Negative Emotion Scale, the lower a youth’s congruence score. This means that the less accepting that mom is of negative emotion, the lower the relation between their youth’s emotional and physical responding. This pattern is reminiscent of the classic pattern of psychophysiological responding typical of avoidantly attached individuals (Diamond, 2001; Gross and Thompson, 2007) wherein they minimize self-reports of distress while demonstrating higher than average levels of physiological distress (Dozier and Kobak, 1992; Roisman et al., 2007). This makes sense when interpreted from within the attachment literature. Children with a secure attachment should feel more comfortable acknowledging their distress. In this situation we would expect that self-reports and physiological reports to display a higher level of congruence. By contrast, a child with an avoidant attachment may minimize their distress and a child with an anxious attachment may maximize their distress, resulting in lower congruence scores.

Unexpectedly, mothers’ acceptance of negative emotion did not predict higher congruence scores. The absence of a relation between these two suggests that parental acceptance of negative emotion may not be the best tool to assess sensitive caregiving. Further analyses showed that the parental acceptance of negative emotion scale was not significantly correlated with mothers’ self-reported maternal warmth. By contrast, parental rejection of negative emotion was significantly correlated with maternal warmth. Specifically, the more rejecting of negative emotion a mother reported being, the less self-reported maternal warmth (*r* = -0.18, *p* = 0.04).

## General Discussion

Although a great deal of research has transpired in the last decade advancing our understanding of interoception, the literature has not considered how interoception develops; and yet, there has been indirect evidence that supports the notion that interoception, a very embodied phenomenon, has social origins. From this perspective, interoception develops initially in the context of interpersonal relationships. To the extent that caregivers recognize, honor, and respect their children’s bodily experiences, the child will develop more accurate interoception. To the extent that a child’s bodily experiences are denied, devalued, ignored, or punished by parents, the child will find ways to avoid feeling them, and develop a distorted sense of interoception.

In this paper we have demonstrated that interpersonal relationships (e.g., attachment styles) are associated with later interoceptive functioning such that when you have an attuned caregiver, you lay better groundwork for future interoception. In study 1 we show that attachment style is linked to interoception generally. We also begin to tease apart some of the more nuanced and interesting ways in which non-attuned caregiving can result in problematic interoception. These results suggest that different types of non-attuned caregiving may result in distinct patterns of interoceptive functioning later in life. While not addressed in this paper, this question will be an important one for future researchers to ask. In study 2 we show these same links, but with a more direct assessment of caregiving and a more direct assessment of interoception. Primarily, in Study 2, we examine the effects of parental rejection of negative emotion. Within the attachment literature this type of dismissive parenting is associated with an avoidant attachment style in children. Why would dismissive parenting be associated with lower interoceptive awareness?

Attachment theorists like Stern (1985) and Fonagy (2001) have argued that for the child to know their own mind, they need to see it reflected in a sensitive caregiver. Here, we contend that for the child to know their own body, they need to see it reflected in a sensitive caregiver also. For example, when a child who is learning to walk falls down and feels physical pain, a parent that acknowledges the child’s discomfort with a statement along the lines of “Ouch! That must have hurt” is arguably promoting greater interoceptive awareness in their child than a parent who exclaims, “You’re fine! That didn’t hurt! Get back up!” The mirroring received by the child in the first instance should allow a child to become confident in their ability to detect bodily cues and comfortable with the acknowledgment and expression of them. This promoting of interoception arises from the parent noticing what the child is experiencing, drawing joint attention to the feeling, and labeling it –processes that can be examined in greater detail in future work considering social antecedents to interoception.

Finally, while this study was unable to examine the neurobiological links between attachment related processes and interoception directly, the extant findings in the literature provide ample evidence that early attachment related experiences, including trauma, shape the neural structure that underlies interoception including the anterior cingulate cortex (van der Werff et al., 2013; Teicher et al., 2014) and the orbitofrontal cortex (Schore, 2005). For example, Schore said in 2005, “The orbitofrontal cortex is the hierarchical apex of the limbic system and is identical to Bowlby’s control system of attachment” (Schore, 2005, p. 216). Thus its functioning is correlated with early caregiving experiences. The orbitofrontal cortex is also the critical brain region for the subjective evaluation of bodily stimuli (Bechara et al., 2000; O’Doherty et al., 2001; Schoenbaum et al., 2003, 2006; Kringelbach, 2005; Rolls and Grabenhorst, 2008). Once bodily cues are felt and noticed, the OFC may be responsible for how an individual interprets them. The OFC also plays an inhibitory role in autonomic functioning, allowing it to be a central player in the process of affect regulation (Fuster, 2001; Schore, 2005). People with an underdeveloped OFC demonstrate greater distress in the face of novel or aversive stimuli (Schore, 2005) and more anxiety related hyperactivation of the interoceptive network. Thus, an underdeveloped OFC typically corresponds with an exaggeration of the importance of bodily cues and the tendency to attribute benign physical cues with deleterious implications.

In closing, we establish the idea that a link exists between attachment related processes and the development of interoception. Attachment related processes are thought to affect the development of interoception by influencing the growth of neural architecture and by modifying functioning of the HPA axis. Further, the idea that caretaking behaviors affect children’s development of interoception is presented. We argue that by continuing to examine the links between social and biological factors, we will begin to build a foundational understanding of how interoception develops.

Future research investigating the relation between interoception and attachment related processes could address the following issues. The first refers to the association between self-reported emotional responding and physiological measures of responding, and the extent to which the congruence between the two can be considered a proxy for interoceptive functioning. The second relates to how early social experiences with a primary caregiver could influence the development of the interoceptive network of the brain. The third focus for future research should be to establish how parenting style, specifically in relation the socialization of bodily cues, could account for variations in interoceptive functioning. Finally, it is of crucial importance in all of this work that we develop a reliable method of quantifying interoception across the lifespan that will facilitate longitudinal developmental studies.

## Ethics Statement

This study was carried out in accordance with the recommendations of the Institutional Review Board at the University of Utah with written informed consent from all subjects. After meeting with study staff, both mothers and youth gave written informed consent in accordance with the Declaration of Helsinki. The protocol was approved by the Institutional Review Board at the University of Utah.

## Author Contributions

KO developed the theoretical idea and performed the analytic calculations. KO, MP, and CW contributed to the final version of the manuscript.

## Conflict of Interest Statement

The authors declare that the research was conducted in the absence of any commercial or financial relationships that could be construed as a potential conflict of interest.
